# Sleeping patterns of Swedish women experiencing a stillbirth between 2000–2014 - an observational study

**DOI:** 10.1186/s12884-016-0982-0

**Published:** 2016-07-28

**Authors:** Ingela Rådestad, Taina Sormunen, Lisa Rudenhed, Karin Pettersson

**Affiliations:** 1 Sophiahemmet University, PB 5605, S-114 86 Stockholm, Sweden; 2 Department of Clinical Science, Intervention and Technology, Karolinska Institutet, Stockholm, Sweden

**Keywords:** Pregnancy, Sleeping position, Stillbirth

## Abstract

**Background:**

External (to the fetus) stressors may act together with maternal factors as well as fetal and placental factors to increase the risk of stillbirth. Data published in 2011 indicate non-left side sleeping positions, particularly the supine one, is such a stressor; we do not know, however, if this new knowledge has influenced the choice of sleeping position among pregnant women.

**Methods:**

Using a web-based questionnaire made available at the home page of the Swedish national infant foundation we collected information on sleeping positions among women who gave birth to a stillborn baby between 2000 and 2014.

**Results:**

The questionnaire was completed by 583 women. About one third of the women reporting their sleeping position stated that they lay down on their the left side when going to bed, and another third reported lying down as often on the left as on the right side. Figures for typically going to bed on the left side the 4 weeks preceding the stillbirth was as follows: 72 (30 %) of 242 between 2011 and 2014 and 86 (27 %) of 313 between 2000 and 2010. Among the 240 women who remembered their position when waking up on the day the stillbirth was diagnosed, 63 (26 %) reported a supine position.

**Conclusion:**

Our data indicate that one third of the women went to bed on the left side the month before the stillbirth. The data are consistent with the notion that efforts in Sweden to advise women to lie on their left side when going to bed may decrease the rate of stillbirth.

## Background

In clinical practice, midwives and obstetricians regularly consider the possibility of occurrence of vena-cava syndrome when, for example, the heart rate of a fetus drops during delivery [1]. The uterus may compress the vena cava, and sometimes also the aorta, when the pregnant woman lies in a supine position [2]. Therefore, the vena-cava syndrome was obvious as a possible explanation when Stacey and co-workers [3] in 2011 reported that a non-left side position during sleep is a risk factor for fetal death [4]. Further, findings in an African population demonstrate that newborns of women who reported supine sleep during pregnancy were at increased risk of low birth weight and stillbirth [5]. We do not know, however, to what extent those and other data may have influenced the sleep positions of pregnant women.

With the evolution of the internet, more and more women can be reached. There certainly are many selection factors determining who goes online, which homepages are visited and who decides to answer a questionnaire made available at a web site [6, 7]. Still, voluntary web-based surveys may give valuable information as long as the particular validity issues are kept in mind when interpreting the data. Moreover, the information sought by researchers may be difficult or impossible to collect by other means. To get an idea on how recently acquired knowledge concerning the etiology of stillbirth may have influenced clinical practice, we collected data through the home page of a Swedish society supporting parents after perinatal loss. The aim of this study was to determine the positions chosen on lying down to sleep by women in Sweden who experienced stillbirth after gestational week 28. An additional aim was to investigate whether the position chosen differed between women giving birth before 2011 and those giving birth after 2011.

## Methods

### Settings and participants

The inclusion criteria were that the women must have given birth to a stillborn baby after gestational week 28 between 2000 and 2014 and had to be able to understand Swedish.

### Data collection

The web-based questionnaire was developed from a previous web-based survey, an interview study [8, 9] and clinical experience. The questions about sleeping position were inspired by Stacey and co-workers [3]. The questionnaire was validated face-to-face with a method similar to the “think aloud” method [10] in a group of women who had experienced stillbirth. In the final version the questionnaire included a total of 87 questions with multiple-choice or open-ended response alternatives.

This study employed the women’s responses to the questions: “What position did you usually take when you went to bed during the last 4 weeks of your pregnancy?”, “What position did you usually take when you went to bed during the final week of your pregnancy?”, “What position did you take when you went to bed the day before you learned that your baby had died?”, “What position were you usually lying in when you woke up during the last 4 weeks of your pregnancy? “What position were you usually lying in when you woke up during the final week of your pregnancy?” and “In what position were you lying when you woke up on the morning of the day you found out that your baby had died?”. Response alternatives were:” Usually on my left side”,“Usually on my right side”, “About as often on one side as the other”, “Usually on my back”, “Usually on my stomach”, “About as often on my stomach as on my back”, “Was sitting up” and “I do not remember”. The reply alternatives for the final night did not contain alternatives”About as often on one side as the other” and “About as often on both sides as on my stomach or back”.

The web-based questionnaire was placed on the homepage of the Swedish National Infant Foundation and answers filed between 1 September 2011 and 31 December 2014, was used in the analysis. The foundation supports parents after perinatal loss and is a member organization of the International Stillbirth Alliance (ISA). The participants were self-recruited after being informed about the study through newspapers, Facebook, and newsletters within the organisation.

### Analysis

Unadjusted risk differences and 95 % confidence intervals based on binominal distribution were calculated to estimate the differences between women with a stillbirth 2000 to 2010 and those with a stillbirth 2011 to 2014. Fisher’s exact test was used when comparing groups.

## Results

Five hundred eighty three (73 %) women fulfilled the inclusion criteria for this study. Sixty-four (11 %) of the women had given birth in gestational week 28–32, 122 (21 %) in gestational week 33–36, and 397 (68 %) in gestational week 37–43. Of the total study population, 335 (57 %) gave birth between 2000 and 2010 and 248 (43 %) between 2011 and 2014. Table 1 shows the sleeping positions at three points in time: 4 weeks, 1 week, and the night preceding the stillbirth. About a third of the women reported going to bed and lying on the left side and another third as often on the left as on the right side. The percentage waking up in the supine position was greater than going to bed for all three time frames asked for: *P* was <0.0001 for the preceding 4 weeks, *P* was <0.0001 for the preceding week and *P* was <0.0001 for the preceding night. Notably a fourth of the women, 63 of 240 accounting for the position, woke up in a supine position the morning before the stillbirth was diagnosed.

**Table 1:** Sleeping positions among 583 women with stillbirth^a^

Position	Went to bed during the last 4 weeks	Woke up during the last 4 weeks	Went to bed during the last week	Woke up during the last week	Went to bed the night before	Woke up the same morning
	N (%)	N (%)	N (%)	N (%)	N (%)	N (%)
Left side	158 (28)	93 (22)	160 (29)	90 (22)	158 (44)	71 (30)
Right side	138 (25)	86 (20)	144 (26)	86 (21)	143 (39)	85 (35)
About as often on one side as other	201 (36)	142 (33)	174 (32)	130 (32)	-	-
Back	30 (5)	85 (20)	32 (6)	77 (19)	38 (10)	63 (26)
Stomach	4 (0.07)	3 (0.7)	3 (0.6)	4 (1)	3 (0.8)	7 (3)
About as often on back as stomach	12 (2)	12 (3)	13 (2)	13 (3)	-	-
Sitting	12 (2)	6 (1.4)	18 (3)	9 (2)	21 (6)	14 (6)
Do not remember	28	156	39	174	220	343

^a^Percentage is calculated based on the number of women who remembered their position

Based on the percentage on the 218 women who remembered both positions, sleeping position when going to bed the night before and woke up position the same day they learned that their baby had died the figures was, Left + Left 56 (26 %), Left + Other 26 (12 %), Other + Left 9 (4 %) and Other + Other 127 (58 %). Women not going to bed or not waking up on the left side were 162 (74 %).

Table 2 shows the sleeping positions during two different periods. No statistically significant differences were noticed when the data for positions taken when going to bed in 2000 to 2010 were compared with the data for 2011 to 2014. Among women who gave birth to a stillborn baby sometime during 2011 to 2014, 72 (30 %) of 242 reported that they had gone to sleep on the left side while the corresponding figures for the years 2000 to 2010 were 86 (27 %) of 313. The absolute difference after rounding is two percent (95 % confidence interval −5 % to 10 %) *P* = 0.57.

**Table 2:** Sleeping position among 335 women with stillbirths in 2000–2010 and 248 women with stillbirths in 2011-2014^a^

Position	2000–2010 Went to bed during the last 4 weeks	2011–2014 Went to bed during the last 4 weeks	2000–2010 Went to bed during the last week	2011–2014 Went to bed during the last week	2000–2010 Went to bed the night before	2011–2014 Went to bed the night before
	N (%)	N (%)	N (%)	N (%)	N (%)	N (%)
Left side	86 (27)	72 (30)	87 (28)	73 (30)	89 (44)	69 (42)
Right side	75 (24)	63 (26)	82 (27)	62 (26)	78 (39)	65 (40)
About as often on one side as other	95 (30)	74 (31)	106 (35)	68 (28)	-	-
Back	14 (4)	16 (7)	15 (5)	17 (7)	22 (11)	16 (10)
Stomach	2 (0.6)	2 (0.8)	1 (0.3)	2 (0.8)	1 (0.5)	2 (1)
About as often on back as stomach	3 (0.09)	9 (4)	5 (2)	8 (3)	-	-
Sitting	6 (0.2)	6 (2)	9 (3)	9 (4)	11 (5)	10 (6)
Do not remember	22	6	30	9	134	86

^a^Percentage is calculated based on the number of women who remembered their sleeping position

## Discussion

Using a unique data set based on reports from women experiencing stillbirth in Sweden between 2000 and 2014, we were able to investigate sleep positions in two groups; one consisting of women giving birth between 2000 and 2010 and the other women giving birth in 2011 and later. We found no convincing data that sleeping positions have changed. A large number of validity issues compromise the comparison between the two periods, as well as a comparison with other data collections. Still, our data leave little doubt that we need to increase our efforts in advising pregnant women to lie down on their left side when going to bed, and, if possible, remain sleeping on the left side to avoid occurrence of vena cava syndrome that may increase the risk of stillbirth.

We do not know what led the women to visit the home page where we posted our questionnaire and, as a second step to answer the questionnaire. We do not know if the factors may differ between the women who gave birth to a child during the first period and the second (first 2000 to 2010, second 2011 to 2014). Thus, one might speculate that a lower percentage of women lay down in a supine position during the second period, and that a higher percentage of those who did so answered our questionnaire. There has been some attention in Swedish media concerning the findings from Stacey and co-workers, which theoretically may have influenced answering patterns [11–13]. But, all questionnaires were completed between 1 September 2011 and 31 December 2014, so memory and selection-induced problems may have occurred among women giving birth before as well as after 2011. There is no routine in Swedish antenatal care to inform about optimal sleeping position. However, we do not know if midwives has taken own initiative to discuss sleeping position with the women.

The time frame certainly is a crucial factor when researchers pose questions about the position taken when going to bed. We cannot observe ourselves sleeping; the correlation between self-reported sleep position and observed sleep position is not perfect [14]. We can ask each woman what position she took when going to bed and what position she was in when she woke up. Moreover, we can also ask about the night before an interview, or what a woman usually did during the previous 4-week period. Formulations of the question as well as answering categories vary between investigations. Memory-induced problems influence results and may depend on the length of the time interval between the stillbirth and the completion of a questionnaire. Thus, we cannot tell to what extent our results reflect the true sleep positions and to what degree the discrepancy between the reported and true sleep position differs between studies.

Stacey and co-workers [3] performed a structured interview carried out 25 days, on average, after a stillbirth. They accounted data for the night before stillbirth or the control interview. Women not lying on their left side when going to bed and not waking up in that position had a relative risk (with 95 % confidence interval) of 2.28 (1.35 to 3.52) of having a stillbirth. Stacey and co-workers classified 72 % women that had a stillbirth as belonging to this group. Our result for the corresponding group is 74 %. Gordon and co-workers [14] interviewed women “as soon as possible” after stillbirth and they were matched by gestation period to the women who experienced stillbirth. They asked the women what their usual sleep position was during pregnancy. They found a relative risk of stillbirth of 5.0 (1.5–16.5) for “supine sleeping position”. They classified 9.7 % who experienced stillbirth as belonging to this group; we found that 5 % went to bed in a supine position and that 20 % woke up in a supine position.

Considering all the limitations concerning our data collection, including that we have no data from women giving birth to a live child, we recognize that our data cannot for certain determine if new knowledge about the possible adverse effects of sleeping positions had any impact on practice in Sweden. Moreover, we lack population data and cannot calculate population attributable fraction of stillbirths in Sweden at the time due to a non-left side sleeping. Further, we had no information about the cause of stillbirth and could consequently not adjust for the cause of the stillbirth. However, our findings about sleeping position may be similar to those reported from New Zealand [3] and Australia [15] and we have no reason to believe relative risks of fetal death in relation to sleep position differ significantly among Sweden, New Zealand and Australia. Consequently we find it reasonable to implement the new knowledge concerning sleeping position in clinical practice and find information channels to help us reach pregnant women more effectively. Ways forward may include actions within the professional organizations of midwives and obstetricians as well as actions from other health authorities. Future studies may show that women who have searched health care websites for information on decreased fetal movements, in particular, should try to sleep all night on the left side, and not in a supine position. Evolving data indicate decreased fetal movements may be an early sign of an increased risk of stillbirth. Certainly maternal factors as well as placental or fetal factors modify to what extent an external stressor, such as a sleep position compressing vena cava, may result in fetal death (triple risk model) [4]. Thus, for certain risk groups sleeping on the left side may be crucial for the birth outcome of a healthy child.

## Conclusions

Our data indicate that one third of the women went to bed on the left side the month before the stillbirth. Efforts in Sweden to advise women to go to sleep and, if possible, continue to sleep on the left side may decrease the rate of stillbirth.

## Abbreviations

ISA, International Stillbirth Alliance
